# Supplementary material for: Merizo: a rapid and accurate protein domain segmentation method using invariant point attention
Source: Nat Commun. 2023 Dec 19;14:8445. doi: 10.1038/s41467-023-43934-4 (PMC10730818; doi:10.1038/s41467-023-43934-4)
Supplement: Supplementary file 3 — Reporting Summary [file 41467_2023_43934_MOESM3_ESM.pdf]

nature portfolio

Corresponding author(s): David T. Jones

Last updated by author(s): Nov 16, 2023

Reporting Summary

Nature Portfolio wishes to improve the reproducibility of the work that we publish. This form provides structure for consistency and transparency in reporting. For further information on Nature Portfolio policies, see our Editorial Policies and the Editorial Policy Checklist.

Statistics

For all statistical analyses, confirm that the following items are present in the figure legend, table legend, main text, or Methods section.

n/a	Confirmed	
☐	☒	The exact sample size (n) for each experimental group/condition, given as a discrete number and unit of measurement
☒	☐	A statement on whether measurements were taken from distinct samples or whether the same sample was measured repeatedly
☒	☐	The statistical test(s) used AND whether they are one- or two-sided<br>*Only common tests should be described solely by name; describe more complex techniques in the Methods section.*
☒	☐	A description of all covariates tested
☒	☐	A description of any assumptions or corrections, such as tests of normality and adjustment for multiple comparisons
☐	☒	A full description of the statistical parameters including central tendency (e.g. means) or other basic estimates (e.g. regression coefficient) AND variation (e.g. standard deviation) or associated estimates of uncertainty (e.g. confidence intervals)
☒	☐	For null hypothesis testing, the test statistic (e.g. F, t, r) with confidence intervals, effect sizes, degrees of freedom and P value noted<br>*Give P values as exact values whenever suitable.*
☒	☐	For Bayesian analysis, information on the choice of priors and Markov chain Monte Carlo settings
☒	☐	For hierarchical and complex designs, identification of the appropriate level for tests and full reporting of outcomes
☒	☐	Estimates of effect sizes (e.g. Cohen's d, Pearson's r), indicating how they were calculated

Our web collection on statistics for biologists contains articles on many of the points above.

Software and code

Policy information about availability of computer code

Data collection	Benchmarking data were collected using softwares:<br><br>UniDoc (https://doi.org/10.1093/bioinformatics/btad070; https://yanglab.nankai.edu.cn/UniDoc/, version date 19/04/2022)<br>SWORD (https://doi.org/10.1126/sciadv.1600552; https://www.dsimb.inserm.fr/sword/, version 1.0)<br>Eguchi-CNN (https://doi.org/10.1093/bioinformatics/btz650; https://github.com/egurapha/prot_domain_segmentor, commit 407ae9f5ff37ae20a32f07dd46b85ef8201659e1)<br>DeepDom (http://www.ncbi.nlm.nih.gov/pmc/articles/pmc6417825/; https://github.com/yuexujiang/DeepDom, commit e479b94a540b9d9a878472c5afd56ff7ba756eed)<br>DPAM (https://doi.org/10.1002/pro.4548; https://github.com/CongLabCode/DPAM, commit 7d1421c956b6bc4fb62cd20263bdfb13aa29cad4).
Data analysis	Python v3.9, PyTorch v1.12.1, SSAP v0.16.10-0-g99edb28, TM-align v20220412, MATLAB 2022b.

For manuscripts utilizing custom algorithms or software that are central to the research but not yet described in published literature, software must be made available to editors and reviewers. We strongly encourage code deposition in a community repository (e.g. GitHub). See the Nature Portfolio guidelines for submitting code & software for further information.

Data

Policy information about availability of data

All manuscripts must include a data availability statement. This statement should provide the following information, where applicable:

- Accession codes, unique identifiers, or web links for publicly available datasets
- A description of any restrictions on data availability
- For clinical datasets or third party data, please ensure that the statement adheres to our policy

Datasets used as well as code developed as part of this study have been deposited to https://github.com/psipred/Merizo. Domain assignments for PDB and AFDB structures from CATH, ECOD, SCOPe and DPAM have been deposited at https://github.com/psipred/Merizo/tree/main/datasets. AlphaFold2 human proteome models used in this study can be downloaded from https://ftp.ebi.ac.uk/pub/databases/alphafold/latest/UP000005640_9606_HUMAN_v4.tar. Protein Data Bank structure files were accessed from https://www.rcsb.org including PDB 3BQC [https://doi.org/10.2210/pdb3BQC/pdb] (protein kinase CK2). Source data are provided with this paper.

Research involving human participants, their data, or biological material

Policy information about studies with human participants or human data. See also policy information about sex, gender (identity/presentation), and sexual orientation and race, ethnicity and racism.

Reporting on sex and gender	This information was not needed and not collected in this study.
Reporting on race, ethnicity, or other socially relevant groupings	This information was not needed and not collected in this study.
Population characteristics	This information was not needed and not collected in this study.
Recruitment	This information was not needed and not collected in this study.
Ethics oversight	This information was not needed and not collected in this study.

Note that full information on the approval of the study protocol must also be provided in the manuscript.

Field-specific reporting

Please select the one below that is the best fit for your research. If you are not sure, read the appropriate sections before making your selection.

[x] Life sciences [ ] Behavioural & social sciences [ ] Ecological, evolutionary & environmental sciences

For a reference copy of the document with all sections, see nature.com/documents/nr-reporting-summary-flat.pdf

Life sciences study design

All studies must disclose on these points even when the disclosure is negative.

Sample size	Sample sizes reported throughout the study were determined based on the availability of training and testing data available. The procedures taken to generate the training and testing split for developing our deep learning method are described clearly in the Methods section "CATH training dataset" and "AFDB models used for fine-tuning".
Data exclusions	No data were excluded from the analyses.
Replication	No attempt at replication was made due to the time and hardware necessary to train the neural network.
Randomization	The experiments were not randomized.
Blinding	The Investigators were not blinded to allocation during experiments and outcome assessment.

Reporting for specific materials, systems and methods

We require information from authors about some types of materials, experimental systems and methods used in many studies. Here, indicate whether each material, system or method listed is relevant to your study. If you are not sure if a list item applies to your research, read the appropriate section before selecting a response.

Materials & experimental systems

n/a	Involved in the study
☒	☐ Antibodies
☒	☐ Eukaryotic cell lines
☒	☐ Palaeontology and archaeology
☒	☐ Animals and other organisms
☒	☐ Clinical data
☒	☐ Dual use research of concern
☒	☐ Plants

Methods

n/a	Involved in the study
☒	☐ ChIP-seq
☒	☐ Flow cytometry
☒	☐ MRI-based neuroimaging

Plants

Seed stocks

Report on the source of all seed stocks or other plant material used. If applicable, state the seed stock centre and catalogue number. If plant specimens were collected from the field, describe the collection location, date and sampling procedures.

Novel plant genotypes

Describe the methods by which all novel plant genotypes were produced. This includes those generated by transgenic approaches, gene editing, chemical/radiation-based mutagenesis and hybridization. For transgenic lines, describe the transformation method, the number of independent lines analyzed and the generation upon which experiments were performed. For gene-edited lines, describe the editor used, the endogenous sequence targeted for editing, the targeting guide RNA sequence (if applicable) and how the editor was applied.

Authentication

Describe any authentication procedures for each seed stock used or novel genotype generated. Describe any experiments used to assess the effect of a mutation and, where applicable, how potential secondary effects (e.g. second site T-DNA insertions, mosiacism, off-target gene editing) were examined.
